# Methyl 5a-acetoxy­methyl-3-isopropyl-8-methyl-1,2,3,3a,4,5,5a,6,7,10,10a,10b-dodeca­hydro-7,10-*endo*-epidioxy­cylohepta­[*e*]indene-3a-carboxyl­ate

**DOI:** 10.1107/S1600536808016474

**Published:** 2008-06-07

**Authors:** Iván Brito, Jorge Bórquez, Luis Alberto Loyola, Alejandro Cárdenas, Matías López-Rodríguez

**Affiliations:** a Departamento de Química, Facultad de Ciencias Básicas, Universidad de Antofagasta, Casilla 170, Antofagasta, Chile; b Departamento de Física, Facultad de Ciencias Básicas, Universidad de Antofagasta, Casilla 170, Antofagasta, Chile; c Instituto de Bio-Orgánica ‘Antonio González’, Universidad de La Laguna, Astrofísico Francisco Sánchez No. 2, La Laguna, Tenerife, Spain

## Abstract

The mol­ecule of the title compound, C_23_H_34_O_6_, is built up from three fused carbocycles, one five-membered, one six-membered and one seven-membered. The five-membered ring has an envelope conformation, whereas the six-membered ring has a perfect chair conformation and the seven-membered ring has a boat conformation. Intra­molecular C—H⋯O hydrogen bonds together with van der Waals inter­actions stabilize the mol­ecular conformation.

## Related literature

For related literature, see: Araya *et al.* (2003 ▶); Cremer & Pople (1975 ▶); Loyola *et al.* (1990 ▶, 2004 ▶); Munizaga & Gunkel (1958 ▶).
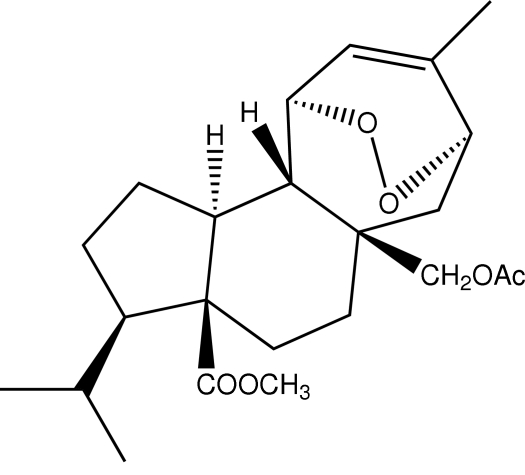


## Experimental

### 

#### Crystal data

C_23_H_34_O_6_
*M* *_r_* = 406.5
Orthorhombic, 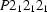

*a* = 7.7014 (1) Å
*b* = 12.1234 (3) Å
*c* = 23.2773 (6) Å
*V* = 2173.34 (8) Å^3^
*Z* = 4
Mo *K*α radiation
μ = 0.09 mm^−1^
*T* = 298 (2) K
0.24 × 0.24 × 0.02 mm

#### Data collection

Nonius KappaCCD area-detector diffractometer
Absorption correction: none
5538 measured reflections
3052 independent reflections
2499 reflections with *I* > 2σ(*I*)
*R*_int_ = 0.031

#### Refinement

*R*[*F* ^2^ > 2σ(*F* ^2^)] = 0.059
*wR*(*F* ^2^) = 0.127
*S* = 1.08
3052 reflections
269 parameters
H-atom parameters constrained
Δρ_max_ = 0.22 e Å^−3^
Δρ_min_ = −0.16 e Å^−3^

### 

Data collection: *COLLECT* (Nonius, 1998 ▶); cell refinement: *DENZO-SMN* (Otwinowski & Minor, 1997 ▶); data reduction: *DENZO-SMN*; program(s) used to solve structure: *SIR97* (Altomare *et al.*, 1999 ▶); program(s) used to refine structure: *SHELXL97* (Sheldrick, 2008 ▶); molecular graphics: *ORTEP-3 for Windows* (Farrugia, 1997 ▶) and *PLATON* (Spek, 2003 ▶); software used to prepare material for publication: *WinGX* (Farrugia, 1999 ▶).

## Supplementary Material

Crystal structure: contains datablocks global, I. DOI: 10.1107/S1600536808016474/bt2719sup1.cif

Structure factors: contains datablocks I. DOI: 10.1107/S1600536808016474/bt2719Isup2.hkl

Additional supplementary materials:  crystallographic information; 3D view; checkCIF report

## Figures and Tables

**Table 1:** Hydrogen-bond geometry (Å, °)

*D*—H⋯*A*	*D*—H	H⋯*A*	*D*⋯*A*	*D*—H⋯*A*
C5—H5*B*⋯O3	0.97	2.39	2.877 (3)	110
C10*A*—H10*A*⋯O3	0.98	2.33	2.848 (3)	112
C10*B*—H10*B*⋯O1	0.98	2.43	2.778 (3)	101

**Fig. 1.**
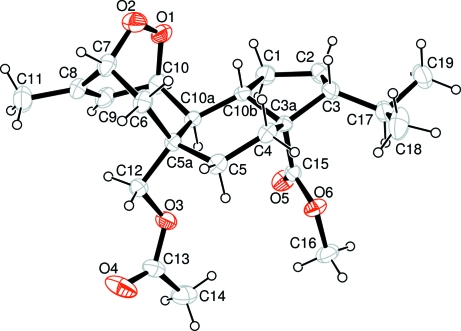
A view of the molecular structure of the title compound, showing the atom-numbering scheme. Displacement ellipsoids are drawn at the 30% probability level and H atoms are shown as small spheres of arbitrary radii.

**Fig. 2.**
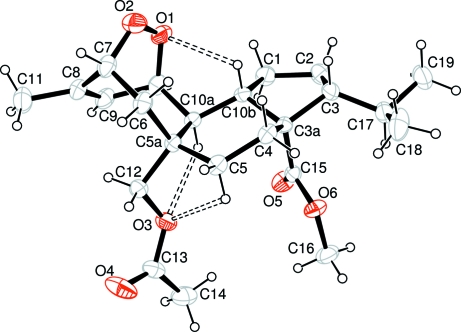
Intramolecular C—H···O interactions (dashed lines) in the title compound.

## supplementary crystallographic information

### Comment

The title compound was obtained from a methylation reaction over the acid group of 17-acetoxymulinic acid, which was previously isolated from Mulinum crassifolium (Apiaceae). Mulinum crassifolium is a 15 cm small shrub, which grows in the north of Chile at altitudes above 4000 m. This plant, commonly known as chuquican, susurco or espinilla is used in the folk medicine, principally against diabetes, and bronchial (caught) and intestinal disorders (Munizaga *et al.*, 1958). Mulinane diterpenes exhibits antiplasmodial (Loyola *et al.*, 2004) and anti-Tripanosomacruzi (Araya *et al.*, 2003) activities.We have undertaken the X-ray crystal-structure determination of (I) in order to establish its molecular conformation and relative stereochemistry. We are not able to determine the absolute stereochemistry by X-ray methods and the configuration shown here was chosen to be in accord with that reported in previous chemical studies (Loyola *et al.*, 1990). The structure consists of a mulinic acid skeleton and the isopropyl, acetyloxymethyl and carboxylate groups at C3, C5a and C3 are β-oriented respectively, whereas the *endo*-peroxide group is α-oriented. The cyclopentane (A), cyclohexane (B) and cycloheptene (C) rings are in an envelope, chair and boat conformation, respectively [Q_2 _= 0.424 (3) Å, φ_2_= 107.2 (4)° for ring A; Q_T_= 0.553 (3) Å, θ = 159.6 (3)°, φ=189.2 (8)° for ring B; Q_T_= 1.123 (3) Å, φ_2_=179.9 (2)°, for ring C] (Cremer & Pople, 1975). The A and B and B and C rings are *trans* and *cis*-fused respectively. The molecular conformation of the title compound, is stabilized by three strong intramolecular hydrogen bonds, Fig.2.

### Experimental

Dried and finely powdered aerial parts of *Mulinum crassifolium* (1530 g) were extracted with petroleum ether at room temperature. The solvent was evaporated in vacuum yielding a gum (40 g). The concentrated petrol ether extract was fractionated on silica gel column with hexane-ethyl acetate mixtures of increasing polarity as elution solvents. The fraction (0.867 g) eluted was further separated and purified by silica gel chromatography to give 120.5 mg of 17-acetoxymulinic acid which was methylated with diazomethane using ethyl ether as solvent at room temperature to give 110 mg de (I). Recrystallization from hexane-ethyl acetate (7:3) at room temperature afforded colourless crystals suitable for X-ray diffraction analysis.

### Refinement

H atoms bonded to C atoms were included in calculated positions and refined as riding atoms, with calculated C - H bond lengths in the range 0.96 - 0.98 Å. For methyl atoms, *U*_iso_(H) = 1.5*U*_eq_(C), while for other H atoms, *U*_iso_(H) = 1.2*U*_eq_(C).

The data are 96% complete. Measurements were nor complete because the single-crystal used was extremely small and curved. The material was difficult to obtain in a suitable crystalline form and the best available specimen was lost late in the data collection. However, the reduced precision does not seriously affect the molecular skeleton and molecular arrangement. We are not able to determine the absolute stereochemistry by X-ray methods and the configuration shown here was chosen to be in accord with that reported in previous chemical studies (Loyola *et al.*, 1990). In the absence of significant anomalous scattering effects, Friedel pairs were averaged. The highest electron-density peak is located 0.71 Å from atom C3a in the final difference Fourier and the deepest hole is located 0.81 Å from O4.

### Crystal data

**Table:**

C_23_H_34_O_6_	*F*_000_ = 880
*M**_r_* = 406.5	*D*_x_ = 1.242 Mg m^−^^3^
Orthorhombic, *P*2_1_2_1_2_1_	Mo *K*α radiation λ = 0.71073 Å
Hall symbol: P 2ac 2ab	Cell parameters from 4314 reflections
*a* = 7.70140 (10) Å	θ = 6.5–28.5º
*b* = 12.1234 (3) Å	µ = 0.09 mm^−^^1^
*c* = 23.2773 (6) Å	*T* = 298 (2) K
*V* = 2173.34 (8) Å^3^	Block, colourless
*Z* = 4	0.24 × 0.24 × 0.02 mm

### Data collection

**Table:**

Nonius KappaCCD area-detector diffractometer	2499 reflections with *I* > 2σ(*I*)
Radiation source: fine-focus sealed tube	*R*_int_ = 0.031
Monochromator: graphite	θ_max_ = 28.7º
φ scans, and ω scans with κ offsets	θ_min_ = 6.5º
Absorption correction: none	*h* = 0→9
5538 measured reflections	*k* = 0→16
3052 independent reflections	*l* = −31→31

### Refinement

**Table:**

Refinement on *F*^2^	H-atom parameters constrained
Least-squares matrix: full	*w* = 1/[σ^2^(*F*_o_^2^) + (0.0421*P*)^2^ + 0.8253*P*] where *P* = (*F*_o_^2^ + 2*F*_c_^2^)/3
*R*[*F*^2^ > 2σ(*F*^2^)] = 0.059	(Δ/σ)_max_ = 0.007
*wR*(*F*^2^) = 0.127	Δρ_max_ = 0.22 e Å^−^^3^
*S* = 1.08	Δρ_min_ = −0.16 e Å^−^^3^
3052 reflections	Extinction correction: none
269 parameters	

### Special details

**Table:**

Geometry. All e.s.d.'s (except the e.s.d. in the dihedral angle between two l.s. planes) are estimated using the full covariance matrix. The cell e.s.d.'s are taken into account individually in the estimation of e.s.d.'s in distances, angles and torsion angles; correlations between e.s.d.'s in cell parameters are only used when they are defined by crystal symmetry. An approximate (isotropic) treatment of cell e.s.d.'s is used for estimating e.s.d.'s involving l.s. planes.
Refinement. Refinement of *F*^2^ against ALL reflections. The weighted *R*-factor *wR* and goodness of fit *S* are based on *F*^2^, conventional *R*-factors *R* are based on *F*, with *F* set to zero for negative *F*^2^. The threshold expression of *F*^2^ > σ(*F*^2^) is used only for calculating *R*-factors(gt) *etc*. and is not relevant to the choice of reflections for refinement. *R*-factors based on *F*^2^ are statistically about twice as large as those based on *F*, and *R*- factors based on ALL data will be even larger.

### Fractional atomic coordinates and isotropic or equivalent isotropic displacement parameters (Å^2^)

**Table:**

	*x*	*y*	*z*	*U*_iso_*/*U*_eq_	
O1	−0.2384 (3)	0.1347 (2)	0.68889 (10)	0.0639 (7)	
O2	−0.1528 (4)	0.2323 (2)	0.71350 (11)	0.0687 (7)	
O3	0.3065 (3)	−0.10383 (17)	0.68850 (9)	0.0518 (5)	
O4	0.5474 (3)	−0.1354 (2)	0.73855 (15)	0.0867 (9)	
O5	0.0901 (3)	−0.14722 (16)	0.55258 (10)	0.0552 (6)	
O6	0.3186 (3)	−0.06177 (16)	0.51523 (9)	0.0487 (5)	
C1	−0.2121 (4)	0.0030 (3)	0.57004 (13)	0.0535 (8)	
H1A	−0.3169	0.039	0.5835	0.052 (2)*	
H1B	−0.2162	−0.0742	0.5808	0.052 (2)*	
C2	−0.1933 (4)	0.0156 (3)	0.50447 (14)	0.0591 (8)	
H2A	−0.2915	0.0558	0.4889	0.052 (2)*	
H2B	−0.1885	−0.0563	0.4863	0.052 (2)*	
C3	−0.0234 (4)	0.0795 (2)	0.49338 (12)	0.0473 (7)	
H3	−0.0524	0.1582	0.4954	0.052 (2)*	
C3A	0.0889 (3)	0.0529 (2)	0.54768 (11)	0.0343 (5)	
C4	0.2315 (4)	0.1336 (2)	0.56491 (12)	0.0398 (6)	
H4A	0.3203	0.136	0.5354	0.052 (2)*	
H4B	0.1834	0.2071	0.5692	0.052 (2)*	
C5	0.3105 (4)	0.0957 (2)	0.62158 (12)	0.0415 (6)	
H5A	0.3964	0.1499	0.6331	0.052 (2)*	
H5B	0.3717	0.0271	0.6146	0.052 (2)*	
C5A	0.1856 (3)	0.0774 (2)	0.67296 (11)	0.0347 (5)	
C6	0.1561 (4)	0.1917 (2)	0.70137 (13)	0.0468 (7)	
H6A	0.2622	0.2115	0.7213	0.052 (2)*	
H6B	0.1402	0.2451	0.6708	0.052 (2)*	
C7	0.0054 (5)	0.2063 (3)	0.74379 (14)	0.0525 (8)	
H7	0.0334	0.2706	0.7676	0.052 (2)*	
C8	−0.0245 (4)	0.1123 (3)	0.78396 (13)	0.0508 (8)	
C9	−0.0977 (4)	0.0257 (3)	0.75995 (13)	0.0520 (8)	
H9	−0.1209	−0.038	0.7808	0.052 (2)*	
C10	−0.1419 (4)	0.0345 (3)	0.69763 (13)	0.0470 (7)	
H10	−0.2224	−0.0261	0.6894	0.052 (2)*	
C10A	0.0111 (3)	0.0217 (2)	0.65466 (11)	0.0337 (5)	
H10A	0.0347	−0.0575	0.6517	0.052 (2)*	
C10B	−0.0497 (3)	0.0591 (2)	0.59511 (11)	0.0356 (6)	
H10B	−0.0792	0.1373	0.599	0.052 (2)*	
C11	0.0302 (6)	0.1227 (4)	0.84575 (14)	0.0737 (11)	
H11A	−0.0064	0.0584	0.8666	0.105 (4)*	
H11B	−0.0224	0.1871	0.8623	0.105 (4)*	
H11C	0.1543	0.1292	0.8478	0.105 (4)*	
C12	0.2888 (4)	0.0041 (3)	0.71470 (13)	0.0462 (7)	
H12A	0.4025	0.0358	0.7218	0.052 (2)*	
H12B	0.228	−0.0019	0.7511	0.052 (2)*	
C13	0.4416 (4)	−0.1652 (3)	0.70431 (15)	0.0500 (8)	
C14	0.4426 (5)	−0.2734 (3)	0.67401 (19)	0.0705 (10)	
H14A	0.5582	−0.2896	0.6611	0.105 (4)*	
H14B	0.3658	−0.2702	0.6416	0.105 (4)*	
H14C	0.4046	−0.3302	0.6998	0.105 (4)*	
C15	0.1611 (3)	−0.0632 (2)	0.53996 (11)	0.0355 (5)	
C16	0.3949 (5)	−0.1677 (3)	0.50295 (16)	0.0618 (9)	
H16A	0.4036	−0.2097	0.5378	0.105 (4)*	
H16B	0.5087	−0.1574	0.4869	0.105 (4)*	
H16C	0.3234	−0.2066	0.4759	0.105 (4)*	
C17	0.0546 (5)	0.0591 (3)	0.43302 (13)	0.0569 (8)	
H17	0.0958	−0.0174	0.4319	0.052 (2)*	
C18	0.2077 (7)	0.1326 (4)	0.41986 (16)	0.0870 (14)	
H18A	0.1763	0.2081	0.4267	0.105 (4)*	
H18B	0.2407	0.1236	0.3804	0.105 (4)*	
H18C	0.3034	0.1129	0.4442	0.105 (4)*	
C19	−0.0846 (7)	0.0713 (4)	0.38666 (15)	0.0855 (14)	
H19A	−0.1338	0.144	0.3886	0.105 (4)*	
H19B	−0.1741	0.0175	0.3928	0.105 (4)*	
H19C	−0.0333	0.0602	0.3495	0.105 (4)*	

### Atomic displacement parameters (Å^2^)

**Table:**

	*U*^11^	*U*^22^	*U*^33^	*U*^12^	*U*^13^	*U*^23^
O1	0.0484 (12)	0.0844 (17)	0.0589 (13)	0.0207 (13)	0.0003 (11)	−0.0204 (13)
O2	0.0731 (16)	0.0593 (15)	0.0738 (16)	0.0192 (14)	0.0024 (14)	0.0054 (13)
O3	0.0489 (12)	0.0487 (11)	0.0579 (12)	0.0085 (10)	−0.0136 (11)	−0.0031 (10)
O4	0.0587 (16)	0.0741 (18)	0.127 (2)	0.0043 (14)	−0.0425 (18)	0.0106 (17)
O5	0.0608 (13)	0.0319 (10)	0.0730 (15)	−0.0098 (10)	0.0136 (12)	−0.0067 (10)
O6	0.0469 (11)	0.0377 (10)	0.0617 (12)	0.0068 (10)	0.0138 (10)	−0.0047 (10)
C1	0.0323 (15)	0.074 (2)	0.0541 (17)	−0.0029 (15)	−0.0033 (13)	−0.0075 (16)
C2	0.0488 (17)	0.076 (2)	0.0520 (17)	0.0034 (17)	−0.0165 (15)	−0.0075 (17)
C3	0.0612 (18)	0.0406 (15)	0.0401 (14)	0.0098 (14)	−0.0074 (14)	−0.0016 (12)
C3A	0.0391 (13)	0.0287 (12)	0.0351 (12)	0.0006 (11)	0.0014 (11)	−0.0003 (10)
C4	0.0450 (15)	0.0311 (13)	0.0435 (14)	−0.0062 (12)	0.0101 (12)	−0.0040 (11)
C5	0.0326 (13)	0.0429 (15)	0.0490 (15)	−0.0102 (12)	0.0035 (12)	−0.0077 (12)
C5A	0.0335 (12)	0.0346 (13)	0.0360 (12)	−0.0055 (11)	−0.0015 (11)	−0.0061 (11)
C6	0.0563 (18)	0.0379 (15)	0.0461 (16)	−0.0087 (14)	0.0008 (14)	−0.0086 (13)
C7	0.070 (2)	0.0420 (16)	0.0460 (16)	−0.0030 (17)	0.0046 (16)	−0.0103 (13)
C8	0.0541 (17)	0.0584 (19)	0.0400 (15)	0.0037 (16)	0.0076 (14)	−0.0049 (14)
C9	0.0612 (19)	0.0476 (16)	0.0471 (16)	−0.0007 (16)	0.0176 (15)	0.0010 (13)
C10	0.0398 (14)	0.0531 (17)	0.0482 (16)	−0.0100 (13)	0.0097 (13)	−0.0049 (14)
C10A	0.0288 (11)	0.0343 (12)	0.0379 (13)	−0.0026 (11)	0.0014 (10)	−0.0047 (10)
C10B	0.0298 (12)	0.0363 (13)	0.0408 (13)	0.0032 (11)	0.0004 (10)	−0.0040 (11)
C11	0.080 (3)	0.097 (3)	0.0439 (18)	0.003 (2)	−0.0008 (19)	−0.0052 (19)
C12	0.0418 (15)	0.0522 (17)	0.0447 (15)	0.0055 (14)	−0.0078 (13)	−0.0073 (13)
C13	0.0363 (15)	0.0521 (18)	0.0615 (19)	0.0016 (13)	0.0014 (14)	0.0189 (15)
C14	0.067 (2)	0.054 (2)	0.090 (3)	0.0167 (19)	0.006 (2)	0.0085 (19)
C15	0.0399 (13)	0.0326 (12)	0.0342 (12)	−0.0005 (12)	−0.0027 (11)	−0.0035 (11)
C16	0.067 (2)	0.0506 (18)	0.068 (2)	0.0200 (17)	0.0143 (19)	−0.0075 (16)
C17	0.084 (2)	0.0468 (16)	0.0399 (15)	0.0123 (19)	−0.0024 (16)	0.0003 (14)
C18	0.123 (4)	0.088 (3)	0.0499 (19)	−0.016 (3)	0.020 (2)	0.005 (2)
C19	0.128 (4)	0.083 (3)	0.0457 (18)	0.032 (3)	−0.021 (2)	−0.0038 (19)

### Geometric parameters (Å, °)

**Table:**

O1—C10	1.438 (4)	C6—H6B	0.97
O1—O2	1.470 (4)	C7—C8	1.492 (5)
O2—C7	1.443 (5)	C7—H7	0.98
O3—C13	1.331 (4)	C8—C9	1.316 (5)
O3—C12	1.450 (4)	C8—C11	1.504 (5)
O4—C13	1.196 (4)	C9—C10	1.494 (5)
O5—C15	1.193 (3)	C9—H9	0.93
O6—C15	1.342 (3)	C10—C10A	1.554 (4)
O6—C16	1.441 (4)	C10—H10	0.98
C1—C10B	1.538 (4)	C10A—C10B	1.532 (4)
C1—C2	1.541 (5)	C10A—H10A	0.98
C1—H1A	0.97	C10B—H10B	0.98
C1—H1B	0.97	C11—H11A	0.96
C2—C3	1.542 (5)	C11—H11B	0.96
C2—H2A	0.97	C11—H11C	0.96
C2—H2B	0.97	C12—H12A	0.97
C3—C17	1.548 (4)	C12—H12B	0.97
C3—C3A	1.565 (4)	C13—C14	1.490 (5)
C3—H3	0.98	C14—H14A	0.96
C3A—C4	1.524 (4)	C14—H14B	0.96
C3A—C15	1.525 (4)	C14—H14C	0.96
C3A—C10B	1.538 (4)	C16—H16A	0.96
C4—C5	1.523 (4)	C16—H16B	0.96
C4—H4A	0.97	C16—H16C	0.96
C4—H4B	0.97	C17—C18	1.510 (6)
C5—C5A	1.551 (4)	C17—C19	1.528 (5)
C5—H5A	0.97	C17—H17	0.98
C5—H5B	0.97	C18—H18A	0.96
C5A—C12	1.538 (4)	C18—H18B	0.96
C5A—C6	1.553 (4)	C18—H18C	0.96
C5A—C10A	1.563 (3)	C19—H19A	0.96
C6—C7	1.534 (5)	C19—H19B	0.96
C6—H6A	0.97	C19—H19C	0.96
			
C10—O1—O2	113.1 (2)	O1—C10—C10A	112.7 (2)
C7—O2—O1	113.1 (2)	C9—C10—C10A	116.4 (2)
C13—O3—C12	117.5 (2)	O1—C10—H10	106.2
C15—O6—C16	116.2 (2)	C9—C10—H10	106.2
C10B—C1—C2	104.8 (3)	C10A—C10—H10	106.2
C10B—C1—H1A	110.8	C10B—C10A—C10	108.7 (2)
C2—C1—H1A	110.8	C10B—C10A—C5A	112.5 (2)
C10B—C1—H1B	110.8	C10—C10A—C5A	115.7 (2)
C2—C1—H1B	110.8	C10B—C10A—H10A	106.5
H1A—C1—H1B	108.9	C10—C10A—H10A	106.5
C1—C2—C3	107.2 (2)	C5A—C10A—H10A	106.5
C1—C2—H2A	110.3	C10A—C10B—C3A	115.0 (2)
C3—C2—H2A	110.3	C10A—C10B—C1	117.5 (2)
C1—C2—H2B	110.3	C3A—C10B—C1	105.7 (2)
C3—C2—H2B	110.3	C10A—C10B—H10B	105.9
H2A—C2—H2B	108.5	C3A—C10B—H10B	105.9
C2—C3—C17	113.6 (3)	C1—C10B—H10B	105.9
C2—C3—C3A	103.3 (2)	C8—C11—H11A	109.5
C17—C3—C3A	119.0 (3)	C8—C11—H11B	109.5
C2—C3—H3	106.7	H11A—C11—H11B	109.5
C17—C3—H3	106.7	C8—C11—H11C	109.5
C3A—C3—H3	106.7	H11A—C11—H11C	109.5
C4—C3A—C15	111.2 (2)	H11B—C11—H11C	109.5
C4—C3A—C10B	106.3 (2)	O3—C12—C5A	107.7 (2)
C15—C3A—C10B	112.5 (2)	O3—C12—H12A	110.2
C4—C3A—C3	118.6 (2)	C5A—C12—H12A	110.2
C15—C3A—C3	107.3 (2)	O3—C12—H12B	110.2
C10B—C3A—C3	100.7 (2)	C5A—C12—H12B	110.2
C5—C4—C3A	108.8 (2)	H12A—C12—H12B	108.5
C5—C4—H4A	109.9	O4—C13—O3	123.3 (3)
C3A—C4—H4A	109.9	O4—C13—C14	125.3 (3)
C5—C4—H4B	109.9	O3—C13—C14	111.4 (3)
C3A—C4—H4B	109.9	C13—C14—H14A	109.5
H4A—C4—H4B	108.3	C13—C14—H14B	109.5
C4—C5—C5A	117.6 (2)	H14A—C14—H14B	109.5
C4—C5—H5A	107.9	C13—C14—H14C	109.5
C5A—C5—H5A	107.9	H14A—C14—H14C	109.5
C4—C5—H5B	107.9	H14B—C14—H14C	109.5
C5A—C5—H5B	107.9	O5—C15—O6	122.1 (3)
H5A—C5—H5B	107.2	O5—C15—C3A	126.3 (2)
C12—C5A—C5	104.5 (2)	O6—C15—C3A	111.6 (2)
C12—C5A—C6	108.8 (2)	O6—C16—H16A	109.5
C5—C5A—C6	106.9 (2)	O6—C16—H16B	109.5
C12—C5A—C10A	111.5 (2)	H16A—C16—H16B	109.5
C5—C5A—C10A	112.6 (2)	O6—C16—H16C	109.5
C6—C5A—C10A	112.1 (2)	H16A—C16—H16C	109.5
C7—C6—C5A	119.2 (2)	H16B—C16—H16C	109.5
C7—C6—H6A	107.5	C18—C17—C19	110.3 (3)
C5A—C6—H6A	107.5	C18—C17—C3	113.1 (3)
C7—C6—H6B	107.5	C19—C17—C3	110.7 (3)
C5A—C6—H6B	107.5	C18—C17—H17	107.5
H6A—C6—H6B	107	C19—C17—H17	107.5
O2—C7—C8	110.1 (3)	C3—C17—H17	107.5
O2—C7—C6	110.4 (2)	C17—C18—H18A	109.5
C8—C7—C6	115.6 (3)	C17—C18—H18B	109.5
O2—C7—H7	106.7	H18A—C18—H18B	109.5
C8—C7—H7	106.7	C17—C18—H18C	109.5
C6—C7—H7	106.7	H18A—C18—H18C	109.5
C9—C8—C7	114.1 (3)	H18B—C18—H18C	109.5
C9—C8—C11	126.4 (3)	C17—C19—H19A	109.5
C7—C8—C11	119.5 (3)	C17—C19—H19B	109.5
C8—C9—C10	117.0 (3)	H19A—C19—H19B	109.5
C8—C9—H9	121.5	C17—C19—H19C	109.5
C10—C9—H9	121.5	H19A—C19—H19C	109.5
O1—C10—C9	108.4 (2)	H19B—C19—H19C	109.5
			
C10—O1—O2—C7	−3.0 (3)	C9—C10—C10A—C5A	−39.5 (4)
C10B—C1—C2—C3	−0.8 (4)	C12—C5A—C10A—C10B	−152.2 (2)
C1—C2—C3—C17	156.3 (3)	C5—C5A—C10A—C10B	−35.1 (3)
C1—C2—C3—C3A	25.9 (3)	C6—C5A—C10A—C10B	85.5 (3)
C2—C3—C3A—C4	−155.8 (2)	C12—C5A—C10A—C10	82.1 (3)
C17—C3—C3A—C4	77.2 (3)	C5—C5A—C10A—C10	−160.9 (2)
C2—C3—C3A—C15	77.4 (3)	C6—C5A—C10A—C10	−40.2 (3)
C17—C3—C3A—C15	−49.7 (3)	C10—C10A—C10B—C3A	179.1 (2)
C2—C3—C3A—C10B	−40.4 (3)	C5A—C10A—C10B—C3A	49.7 (3)
C17—C3—C3A—C10B	−167.5 (3)	C10—C10A—C10B—C1	−55.4 (3)
C15—C3A—C4—C5	−60.2 (3)	C5A—C10A—C10B—C1	175.1 (2)
C10B—C3A—C4—C5	62.4 (3)	C4—C3A—C10B—C10A	−63.6 (3)
C3—C3A—C4—C5	174.8 (2)	C15—C3A—C10B—C10A	58.3 (3)
C3A—C4—C5—C5A	−54.7 (3)	C3—C3A—C10B—C10A	172.2 (2)
C4—C5—C5A—C12	161.1 (2)	C4—C3A—C10B—C1	165.1 (2)
C4—C5—C5A—C6	−83.6 (3)	C15—C3A—C10B—C1	−73.0 (3)
C4—C5—C5A—C10A	39.9 (3)	C3—C3A—C10B—C1	40.9 (3)
C12—C5A—C6—C7	−82.6 (3)	C2—C1—C10B—C10A	−155.3 (3)
C5—C5A—C6—C7	165.1 (3)	C2—C1—C10B—C3A	−25.3 (3)
C10A—C5A—C6—C7	41.2 (4)	C13—O3—C12—C5A	154.3 (2)
O1—O2—C7—C8	−48.3 (3)	C5—C5A—C12—O3	−69.1 (3)
O1—O2—C7—C6	80.6 (3)	C6—C5A—C12—O3	177.0 (2)
C5A—C6—C7—O2	−85.8 (3)	C10A—C5A—C12—O3	52.8 (3)
C5A—C6—C7—C8	40.0 (4)	C12—O3—C13—O4	−0.2 (5)
O2—C7—C8—C9	51.4 (4)	C12—O3—C13—C14	179.9 (3)
C6—C7—C8—C9	−74.6 (4)	C16—O6—C15—O5	2.0 (4)
O2—C7—C8—C11	−129.2 (3)	C16—O6—C15—C3A	−176.7 (3)
C6—C7—C8—C11	104.8 (4)	C4—C3A—C15—O5	143.0 (3)
C7—C8—C9—C10	−1.0 (4)	C10B—C3A—C15—O5	23.9 (4)
C11—C8—C9—C10	179.7 (3)	C3—C3A—C15—O5	−85.9 (3)
O2—O1—C10—C9	51.2 (3)	C4—C3A—C15—O6	−38.5 (3)
O2—O1—C10—C10A	−79.1 (3)	C10B—C3A—C15—O6	−157.5 (2)
C8—C9—C10—O1	−50.8 (4)	C3—C3A—C15—O6	92.6 (3)
C8—C9—C10—C10A	77.4 (4)	C2—C3—C17—C18	172.8 (3)
O1—C10—C10A—C10B	−41.0 (3)	C3A—C3—C17—C18	−65.2 (4)
C9—C10—C10A—C10B	−167.1 (3)	C2—C3—C17—C19	48.4 (4)
O1—C10—C10A—C5A	86.6 (3)	C3A—C3—C17—C19	170.4 (3)

### Hydrogen-bond geometry (Å, °)

**Table:**

*D*—H···*A*	*D*—H	H···*A*	*D*···*A*	*D*—H···*A*
C5—H5B···O3	0.97	2.39	2.877 (3)	110
C10A—H10A···O3	0.98	2.33	2.848 (3)	112
C10B—H10B···O1	0.98	2.43	2.778 (3)	101
